# Stimulating growth, root quality, and yield of carrots cultivated under full and limited irrigation levels by humic and potassium applications

**DOI:** 10.1038/s41598-023-41488-5

**Published:** 2023-08-31

**Authors:** Ayman M. S. Elshamly, Saad M. A. Nassar

**Affiliations:** 1 Water Studies and Research Complex, National Water Research Center, Cairo, Egypt; 2 Department of Genetic Resources, Desert Research Center, El-Matareya, Cairo, Egypt

**Keywords:** Plant reproduction, Plant stress responses, Climate sciences, Environmental sciences

## Abstract

Water stress poses a significant challenge for carrot cultivation, leading to decreased yield and inefficient water use efficiency. Therefore, it is crucial to provide plants with suitable supplements that enhance their stress resistance. In this study, we investigated the effectiveness of humic and potassium applications on carrot growth, yield characteristics, root quality, and water use efficiency under varying irrigation levels. A split-split plot experiment was conducted, with two levels of gross water requirements (GWR) (100% and 80%) assigned to the main plots. The subplots were treated with humic acid through foliar application (Hsp) or soil drenching (Hgd). The sub-subplots were further divided to assess the impact of foliar potassium sources (potassium humate, Kh) and mineral applications (potassium sulfate, K_2_SO_4_). The results revealed a substantial reduction in carrot yield under limited irrigation, reaching about 32.2% lower than under GWR100%. Therefore, under limited irrigation conditions, the combined application of Hgd and K_2_SO_4_ resulted in a significant yield increase of 78.9% compared to the control under GWR80%. Conversely, under GWR100%, the highest average yield was achieved by applying either Hsp and Kh or Hsp and K_2_SO_4_, resulting in yields of 35,833 kg ha^−1^ and 40,183 kg ha^−1^, respectively. However, the combination of Hgd and Kh negatively affected the yield under both GWR100% and GWR80%. Nonetheless, applying Kh in combination with Hgd under GWR80% led to improved nitrogen, phosphorus, potassium, potassium/sodium ratio, and total sugar concentrations, while reducing sodium content in carrot roots. Based on this study, it is recommended to adopt GWR80% and treat plants with a combination of Hgd and foliar K_2_SO_4_. This approach can help plants overcome the negative effects of water stress, improve yield and root quality, and achieve optimal water use efficiency.

## Introduction

Climate change, rapid population growth, and soil degradation pose significant challenges to the agriculture sector^1^. Addressing these challenges requires the development of water-saving agricultural practices and improvements in water use efficiency to ensure global food security^2^. Water stress adversely affects nutrient balance, primary and secondary metabolism, and turgor regulation in plants^3,4^ resulting in reduced crop yield and quality^5^. To mitigate the negative impacts of water stress, the use of organic materials has been considered as an agronomic solution^6^.

Humic acid, a key component of plant nutrition, is widely used to supplement synthetic and organic fertilizers. It is a naturally occurring polymeric-heterocyclic organic compound that contains carboxylic (COOH^−^), phenolic (OH^−^), alcoholic, and carbonyl fractions^7,8^. Humic substances are generally classified into humic acids, fulvic acids, and humins^9^. While humins have a non-degrading fraction and have received less attention, researchers have focused on humic and fulvic acids due to their ability to rapidly improve soil fertility and health^10^. Numerous studies have reported that humic acid applications offer several benefits, including enhanced plant growth, cell permeability, photosynthetic rate, cell elongation, soil structure, water use efficiency, and nutrient transport and availability^11–15^. Furthermore, humic acid has been found to promote root growth and nutrient absorption, making it an excellent foliar fertilizer that positively influences leaf, root, and fruit development^16–18^.

Given these properties, the adoption of humic acid in carrot plants is expected to increase productivity and improve crop quality. Carrot roots (*Daucus carota* L.) are an important vegetable known for their high content of carbohydrates, carotene, minerals, fiber, and vitamin C^19,20^. However, conflicting results have been reported in the literature regarding the effects of humic acid application on plant growth and yield^21,22^, Some studies found no significant effects, while others observed negative effects on yield traits and quality^23,24^. In this concern, Ampong et^10^ demonstrated that it is essential to test the effects of humic acid under specific conditions before reliable recommendations can be made. Therefore, we hypothesized that the adoption of potassium applications could lead to a kind of nutritional balance with humic, improving yield quality, in particular under water stress conditions.

In this regard, potassium applications play a crucial role in plant-water relations as they catalyze metabolic functions, promote starch transformation into soluble sugars, enhance enzyme activity, and improve protein, carbohydrate, and fat synthesis. Potassium applications also contribute to the translocation of photosynthetic products and enhance plants' ability to resist pests, diseases, and environmental stress^25,26^. Moreover, they have a positive impact on preventing and mitigating the adverse effects of sodium toxicity by modulating the antioxidant system^27^. The combined application of humic acid and mineral fertilizers forms complexes that release nutrients slowly, facilitating nutrient uptake. The interaction effects between humic acid and potassium applications depend on the source of humic acid, application rate, and crop type^28^.

While previous studies have examined the positive effects of combined potassium and humic acid applications on plant growth and yield in crops such as potatoes, sugar beet, wheat, peanuts, and peas^16,29–32^ few studies have investigated their combined influences on carrot plant growth and yield.

Therefore, the application of humic on carrot plants was investigated. Whereas that humic usage not generating an environmental advertising impact. In addition, it can also play a vital role in enhancing yield and can be used as a substance of natural origin to decrease the use of chemical fertilizers. On the other hand, little information is available regarding its effects as a sole and combined application with potassium sources on the carrot yield and water use efficiency under two irrigation levels. Thus, their applications in different methods and enrichment with different potassium substances can have different impacts which have not been previously investigated in such an approach and thus contribute to the novelty of this study.

## Materials and methods

### Experimental site and growth conditions

An open field experiment was conducted at the experimental farm of the National Water Research Center's water studies and research complex station, Egypt, Aswan, Toshka city, which is located in the southern of Egypt at the latitude of 22°, 24`0.11` N longitude of 31°,35`0.43` E and of altitude 188 m. The experiment was implemented through two successive winter seasons of 2019/2020 and 2020/2021, to study the effect of humic and potassium applications under full and limited irrigation on nitrogen (N), phosphorus (P), potassium (K), yield and water use efficiency (Iwue) of carrot plants. The studied area lies in an arid climatic province, Table 1 presented the averages of meteorological data, which have been collected from the Toshka weather station during the growing seasons. The main source of irrigation water is groundwater through a well that was dug in the studied area. Irrigation water samples were collected during cultivation three times (before, mid of the growing season, and at harvest) for the analysis. The average of the chemical properties of these samples during the two seasons of 2019/2020 and 2020/2021 are given in Table 2. Regarding the water quality as mentioned by Zaman et al.^33^, it was classified as C_2_S_1_. According to USDA Soil Survey Staff^34^, the selected soil has a loamy sand texture. While the remaining physical and chemical properties of the experimental soil are given in Table 3. All physical and chemical of soil and irrigation water are determined by following standardized methods Zaman et al.^33^, Estefan et al.^35^, and Vaz et al.^36^.

**Table 1:** Average weather parameters obtained from the local weather station (Toshka station) for the two growing seasons of 2019/2020 and 2020/2021.

Month	RH		Air temperature		Soil temperature		WS	AP	P
	RH _max_	RH_min_	Tmax	Tmin	Tmax	Tmin			
November	55.0	18.3	32.1	17.4	28.9	22.6	2.8	994.7	0
December	57.3	22.6	25.6	13.8	26.8	21.3	4.0	994.2	0
January	59.8	23.3	24.0	8.43	24.4	17.7	2.9	999.0	0
February	50.2	16.6	25.0	8.3	27.4	20.7	3.5	996.5	0
March	37.0	7.2	29.9	13.9	29.6	22.2	3.0	991.9	0

RH average relative humidity (%), RH_max_ average maximum relative humidity, RH_min_ average minimum relative humidity, T_max_ maximum temperature (°C), T_min_ minimum temperature (°C), WS wind speed (meter second^−1^), AP atmospheric pressure (millibars), and P precipitation (mm).

**Table 2:** Average water chemical properties at the experimental site during the two growing seasons of 2019/2020 and 2020/2021.

Parameter	Unit	Values	Reference
pH		6.32	Estefan et al.^35^
TDS	mg L^−1^	646	
Electrical conductivity (EC)	mg L^−1^	1.01	
HCO_3_	mg L^−1^	73.2	
Calcium cations (Ca^+2^)	mg L^−1^	64.1	
Magnesium cations (Mg^+2^)	mg L^−1^	15.8	
Sodium cations (Na^+^)	mg L^−1^	117.3	
Potassium cations (K^+^)	mg L^−1^	4.7	
Chloride anions (Cl^−^)	mg L^−1^	113.5	
Sulfate anions (SO_4_^−2^)	mg L^−1^	240.2	
SAR		3.40	Zaman et al.^33^
RSC		− 3.3	

TDS: total dissolved solids; RSC: the residual sodium carbonate; SAR: the sodium adsorption ratio. Each value represents the mean of three replications.

**Table 3:** Average values of the physicochemical properties of the soil before the experimental initiated during the growing seasons of 2019/2020 and 2020/2021.

Parameter	Unit	Value		Analytical method used
		0–30	30–60	
Mechanical analysis				
Sand	%	86.8	88.7	Soil Survey Staff^34^, Estefan et al.^35^
Silt	%	3.95	4.15	
Clay	%	9.25	7.15	
Texture	Loamy sand			
Chemical analysis				
pH (1:2.5)		7.92	7.93	Estefan et al.^35^
Electrical conductivity (EC)	ds m^−1^	1.69	0.86	
CaCO_3_	%	1.69	1.56	
Calcium cations (Ca)	mg kg^−1^	242.5	121.5	
Available Phosphorus (P)	mg kg^−1^	7.0	6.0	
Available Potassium (K)	mg kg^−1^	38.0	19.0	
Magnesium cations (Mg)	mg kg^−1^	109.0	51.0	
Sodium cations (Na)	mg kg^−1^	704.0	346.0	
Chloride anions (Cl)	mg kg^−1^	678.0	339.0	
Bicarbonate anions (HCO_3_)	mg kg^−1^	173.0	90.0	
Sulfate anions (SO_4_)	mg kg^−1^	237.0	115.0	
Organic matter	%	0.01	0.1	
Water status				
Saturation percent	%	27.0	25.3	Vaz et al.^36^
Field capacity	%	13.0	11.0	
Wilting point	%	4.5	3.5	

Each value represents the mean of three replications.

### Experimental details

In order to achieve the objective of the current study, a split-split plot design was used with three replicates. In the main plots, two irrigation water levels were allocated, i.e., 100 and 80% GWR for carrots. In this concern, before the experiment began, soil water parameters were measured, and allowed for a reduction in soil moisture to 60% of the available water, which was the critical limit on carrot development based on previous studies. Therefore, based on this knowledge, irrigation is carried out every two days. Moreover, the irrigation amounts applied to carrot plants under 80% GWR level were proportionally obtained from 100% GWR, as will be handled later. A set sprinkler irrigation system was used to irrigate carrots and each irrigation plot was equipped with a manometer valve to maintain the operating pressure at 2.5 bar and a flow emitter which was used to control the quantity of the targeted irrigation water at each irrigation level. The distance between the sprinklers was 10 m, and the distance between the lines was 9 m. Furthermore, there were buffer zones between the experimental units to avoid interactions (9 m width). While humic (H) application methods were assigned in the subplots (control, Hgd, and Hsp). In the Hgd treatment, H was applied at a rate of 30 L ha^−1^ as soil applications in three equal portions initiated after 30 days of cultivation, 60 and 90 days. On the other hand, Hsp took the same previous dates for applying H as foliar applications at a rate of 3 g L^−1^. While, sub-sub plots were divided into three parts to apply K applications, namely (control, Kh, and K_2_SO_4_). In the control treatments, carrot plants were sprayed with distilled water. While in Kh and K_2_SO_4_ treatments, 2 g L^−1^ of K as foliar spraying was applied in a Kh and K_2_SO_4_ form, initiated after 30 days from cultivation then four times every 15 days interval. The net space (10.0 m long × 4.5 m width) of each experimental unit, accordingly, the experimental work involved 54 plots {2 irrigations levels × 3 H treatments × 3 K treatments × 3 replicates}.

### Agronomic practices

Seeds of carrot (*Daucus carota* L., cv. Kuroda Max), were sown on the 2 of November 2019 and the 4 of November 2020 in the first and second seasons, respectively. The experimental site was well prepared and soil tillage. All agricultural practices required for carrot production were followed as commonly used in the region and the Egyptian Ministry of Agriculture recommendations for newly reclaimed soil. Carrot seeds were sown on ridges, with a 75 cm spacing between rows and 8 cm between plants. Carrot seeds were purchased from Takii Seed Co. This cultivar is recommended as a highly yielding-commercial cultivar. Furthermore, this cultivar and the implemented methods in the current study complied with international, national, and institutional guidelines and legislation. The harvest was on the 29 of February 2020 and 5 of March 2021, with a total growing season of 120 and 122 days during the first and second seasons, respectively.

**H and Kh properties:** H was purchased from Egyptian Canadian for humate Co, H products had 65.0% humic substances (involving 13.0% active H and 3% fulvic acid), and 5.0% potassium. While KH was purchased from Zain Fert Co. (it contains 75% H + 4% fulvic acid + 2% iron (Fe) + 10% K_2_O).

### Calculations related to irrigation

#### Calculations of reference evapotranspiration

The reference evapotranspiration (ETo) was determined by directly entering specific data that were obtained from the Toshka agrometeorological station, in the CROPWAT package, version 8.0, and ETo was calculated by Penman–Monteith equation as indicated by^37^, which can be calculated as:

1 $${\text{ETo}} = \frac{{0.408{\Delta }\left( {{\text{Rn}} - {\text{G}}} \right) + {\gamma }\frac{900}{{T + 273}}{\text{U}}2{ }\left( {{\text{es}} - {\text{ea}}} \right){ }}}{{{\Delta } + {\gamma }\left( {1 + 0.34{\text{U}}2} \right)}}$$

where:

ETo = Reference evapotranspiration (mm day^−1^).

Rn = Net radiation (MJm^−2^d^−1^).

G = Soil heat flux (MJm^−2^d^−1^).

Δ = Slope vapor pressure and temperature curve (kPa ^o^C^−1^).

γ = Psychrometric constant (kPa °C^−1^).

U2 = Wind speed at 2 m height (ms^−1^).

es-ea = Vapor pressure deficit (kPa).

T = Mean daily air temperature at 2 m height (°C).

#### Calculations of crop evapotranspiration

The crop evapotranspiration of carrot (ETc) was calculated according to^38^ as the following equation:

2 $${\text{ETc }} = \, \left( {{\text{ETo }} \times {\text{ Kc stages}}} \right)$$

where.

ETc = Crop evapotranspiration (mm day^−1^).

ETo = Reference evapotranspiration (mm day^−1^).

Kc = Crop coefficient (which was equaled 0.7, 1.05, and 0.95 for Kc _mid_, Kc _mid_, and Kc _end_ according to^39^.

#### Calculations of water requirement

Then the GWR_100_ was calculated according to^40,41^

3 $${\text{GWR}} = \frac{{{\text{ETc}} \times Se}}{{{\text{E}}a \times \left( {1 - LR} \right)}} \times 10$$

where.

GWR = The gross water requirement (m^3^ ha^−1^).

Se = The evapotranspiration area percentage.

LR = Leaching water requirement 10 %.

Ea = Irrigation system efficiency, 0.78.

Etc = Crop evapotranspiration (m^3^).

Accordingly, during the two growing seasons of 2019/2020 and 2020/2021, the average total seasonal amounts of GWR were 9660 and 5785 m^3^ ha^−1^ for the GWR100 and GWR80, respectively.

### The chemical measurements

After the harvest (with an average of 121 days), the carrot roots were chopped into small pieces, air-dried, and oven dried at 70°C for five days then ground using stainless steel equipment. Additionally, soil samples were taken from the root ridges in each treatment to measure (total dissolved solids, available K, and organic matter) according to Estefan et al.^35^, while the macronutrients {N, P, K, calcium (Ca)}, and sodium (Na) in carrot roots were estimated according to^42,43^. The total carotenoids and carbohydrates (%) in the roots were determined according to the method described by Boadi et al.^44^, total sugar (%) was determined according to the method described by Yusuf et al.^45^. While the total chlorophyll in leaves at harvest was determined according to Molina et al.^46^.

### Yield and yield components

At the harvest, ten plants were randomly taken from each plot to record the average of the following measurements were recorded: plant height (cm), number of leaves, root length and root diameter (cm), leaves plant fresh weight (g), weight of root (g), and root yield (kg m^−2^) were recorded for each plot and then converted to kg ha^−1^.

### The calculation of Iwue

The Iwue was calculated as the following equation of^47^

$${\text{Iwue}} = { }\left( {\frac{{\text{Y}}}{{\text{GWR }}}} \right)$$

where.

Iwue = irrigation water use efficiency (kg m^−3^).

Y = Yield (kg ha^−1^) and.

GWR = The gross water requirement (m^3^ ha^−1^).

### Statistical analysis

Means of variance (ANOVA) were analyzed in all data to determine any statistically significant differences. Statistical analysis was determined by using the statistical package Costat version 6.303. The least significant difference (LSD) for the average data of the two growing seasons was used to test the differences between treatments (at the *p* ≤ 0.05 level) as per^48^.

### Ethical approval and consent to participate

This manuscript is an original paper and has not been published in other journals. The authors agreed to keep the copyright rule.

## Results

### The analysis of (TDS, available K, and organic matter) in the soil at the end of the experiment

Data of the average (TDS, available K, and organic matter) values in the soil under two levels of gross water requirements (100 and 80%) at the end of the experiment are tabulated in Table 4. While Table 5 showed the analysis of variance results for individual and interaction impacts on the investigated parameters. In general, by comparing the impacts of examined treatments on TDS, by adopting the examined treatments, there were significant differences in TDS values. Likewise for available K, where the results showed that there were no significant differences in available K due to irrigation levels, while there were significant differences as a consequence of the other individual and combined interactions. Also, the statical analysis demonstrated that there were no significant differences in available K due to irrigation levels, the combined interaction of irrigation × K sources, or due to the combined interaction of irrigation × H applications on organic matter; contrary to the remaining interactions.

**Table 4:** Average values of (TDS, available K, and organic matter) analysis of the soil under two levels of gross water requirements (100 and 80%) at the end of the experiment during the seasons of 2019/2020 and 2020/2021.

The targeted treatments	The analysis	Unit	Value		Analytical method used
			100%	80%	
Control	TDS	mg L^−1^	1259 e	1445 a	Estefan et al.^35^
	Available Potassium (K)	mg kg^−1^	48.7 a	39.3 bc	
	Organic matter	%	0.03 c	0.01 cd	
Kh	TDS	mg L^−1^	1231 ef	1433 a	
	Available Potassium (K)	mg kg^−1^	38.3 bc	36.0 bc	
	Organic matter	%	0.05 c	0.02 d	
K_2_SO_4_	TDS	mg L^−1^	1245 ef	1441 a	
	Available Potassium (K)	mg kg^−1^	42.3 ab	38.0 bc	
	Organic matter	%	0.04 c	0.01 e	
Hgd	TDS	mg L^−1^	1218 f.	1375 b	
	Available Potassium (K)	mg kg^−1^	33.0 c	32.2 c	
	Organic matter	%	0.07 ab	0.04 c	
Hgd + Kh	TDS	mg L^−1^	1198 f.	1286 d	
	Available Potassium (K)	mg kg^−1^	28.0 d	27.5 d	
	Organic matter	%	0.08 a	0.06 bc	
Hgd + K_2_SO_4_	TDS	mg L^−1^	1225 ef	1320 c	
	Available Potassium (K)	mg kg^−1^	36.0 c	29.2 d	
	Organic matter	%	0.06 bc	0.05 bc	
Hsp	TDS	mg L^−1^	1192 f.	1416 b	
	Available Potassium (K)	mg kg^−1^	28.0 de	36.7 c	
	Organic matter	%	0.08 a	0.04 c	
Hsp + Kh	TDS	mg L^−1^	1176 f.	1383 b	
	Available Potassium (K)	mg kg^−1^	23.0 e	33.8 c	
	Organic matter	%	0.05 c	0.01 e	
Hsp + K_2_SO_4_	TDS	mg L^−1^	1186 ef	1398 b	
	Available Potassium (K)	mg kg^−1^	27.3 d	35.0 c	
	Organic matter	%	0.04 c	0.03 cd	

TDS: total dissolved solids; Control: spray with pure water; Hgd: applying humic acid as soil application; Hsp: applying humic acid as foliar spray applications; Kh: applying potassium humate as foliar spray applications; K_2_SO_4_: applying potassium sulfate as foliar spray applications; 100%: applying 100% of gross irrigation water requirements; 80%: applying 80% of gross irrigation water requirements. Each value represents the mean of three replications.

**Table 5:** Variance analysis of the investigated parameters.

Source of variation	df	PH	LFW	NL	RY	RW	RD	RL
Gross water requirements levels (GWR)	1	*	*	*	*	NS	NS	*
Humic applications (H)	2	*	*	*	*	*	*	*
Potassium applications (K)	2	*	*	*	*	*	*	*
GWR × H	2	NS	*	*	*	*	NS	NS
GWR × K	2	NS	*	*	*	NS	NS	NS
H × K	4	*	*	NS	*	*	*	*
GWR × H × K	24	*	*	*	*	*	*	*

		CA	CHL	TS	CAR	N	P	K
Gross water requirements levels (GWR)	1	*	*	*	*	*	*	*
Humic applications (H)	2	*	*	NS	*	*	*	*
Potassium applications (K)	2	*	*	*	*	*	*	*
GWR × H	2	NS	*	*	NS	*	*	NS
GWR × K	2	*	NS	NS	*	*	*	*
H × K	4	*	*	*	NS	*	*	*
GWR × H × K	24	*	*	*	*	*	*	*

		Ca	Na	Ca/Na	K/Na	TDS	OM	AKS
Gross water requirements levels (GWR)	1	*	*	NS	NS	*	NS	NS
Humic applications (H)	2	*	*	*	*	*	*	*
Potassium applications (K)	2	*	*	*	*	*	*	*
GWR × H	2	NS	*	*	*	*	*	*
GWR × K	2	NS	NS	NS	NS	*	NS	*
H × K	4	*	*	*	*	*	NS	*
GWR × H × K	24	*	*	*	*	*	*	*

PH: plant height; LFW: leaves fresh weight; NL: number of leaves; RY: root yield; RW: roots weight; RD: root diameter; RL: root length; CA: carbohydrates; CHL: total chlorophyll; TS: total sugar; CAR: carotenoids; N: nitrogen; P: phosphorus; K: potassium; Ca: calcium; Na: sodium; TDS: total dissolved solids; OM: organic matter; AKS: available potassium in soil; NS: non-significance; * significance at *P* ≤ 0.05.

Relative to full irrigation (GWR100%), TDS of the control treatment fell by 2.2, 1.1, 3.2, 4.9, 2.7, 5.3, and 6.7% for Kh, K_2_SO_4_, Hgd, Hgd + Kh, Hgd + K_2_SO_4_, Hsp, Hsp + Kh, Hsp + K_2_SO_4_, respectively. Likewise, available K in the soil samples decreased by 21.4% (Kh), 13.1% (K_2_SO_4_), 32.2% (Hgd), 42.5% (Hgd + Kh), 26.1% (Hgd + K_2_SO_4_), 42.5% (Hsp), 52.8 (Hsp + Kh), and 43.9% (Hsp + K_2_SO_4_). Finally, relative to full irrigation (GWR100%), soil organic matter in the control treatment attained a greater increase by 266.7, 233.3, and 200% for Hgd + Kh, Hgd, and Hgd + K_2_SO_4_, respectively**.**

On the other hand, relative to limited irrigation (GWR80%), TDS of the control treatment fell by 0.8, 0.3, 4.8, 11.0, 8.7, 2.0, and 4.3% for Kh, K_2_SO_4_, Hgd, Hgd + Kh, Hgd + K_2_SO_4_, Hsp, Hsp + Kh, Hsp + K_2_SO_4_, respectively. Likewise, available K in the soil samples decreased by 8.3% (Kh), 3.3% (K_2_SO_4_), 18.1% (Hgd), 30.0% (Hgd + Kh), 25.7% (Hgd + K_2_SO_4_), 6.6% (Hsp), 14.0 (Hsp + Kh), and 10.9% (Hsp + K_2_SO_4_). Finally, relative to full irrigation (GWR100%), soil organic matter in control treatment was attained the greater increase by 600 and 500% for Hgd + Kh and Hgd + K_2_SO_4_, respectively**.**

TDS levels attained higher increases by 18.8% (Hsp) 17.6% (Hsp + Kh) and 16.4% (Kh) under limited irrigation conditions (GWR80%), respectively, compared with those under well-watered conditions. On the other side, available K in the soil samples achieved fluctuating results, where it attained greater increases values at (Hsp + Kh) and (Hsp) under limited irrigation conditions by 47.0% and 31.1%, respectively, compared with those under well-watered conditions. Similarly, soil organic matter either achieved fluctuating results, where it attained greater decreases values at (Hsp) and (Hsp + Kh) under limited irrigation conditions by 50.2% and 50.0%, respectively, compared with those under full irrigation (GWR100%).

### Impacts of the H and K applications on (N, P, K, Ca, Na, Ca/ Na ratio, and K/ Na ratio) of the carrot roots under full and limited irrigation levels

**Figure 1:**
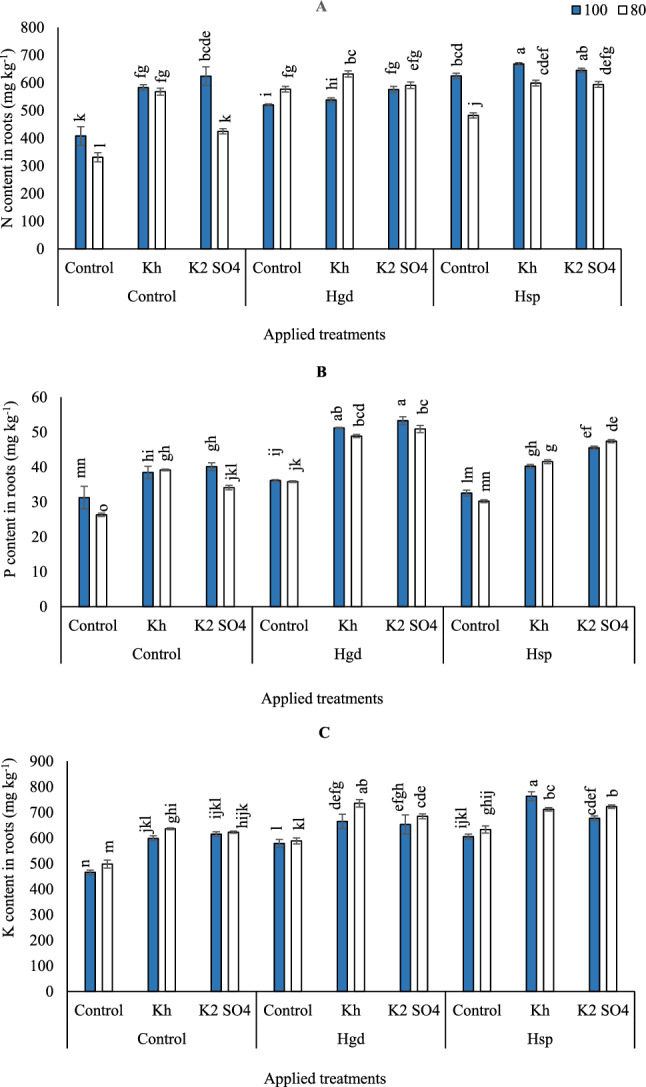
Influence of the sole or combined application of humic acid & potassium sources in the carrot plants under full and limited irrigation levels on N- Nitrogen (**A**), P- Phosphorus (**B**), and K- Potassium (**C**). The illustrated values in the figures are the average of the two growing seasons of 2019/2020 and 2020/2021. Vertical bars represent ± standard error (SE) of the means. Bars with different letters are statistically significant at *p* ≤ 0.05. Abbreviations: Control (spray with pure water); Hgd (applying humic acid as soil application); Hsp (applying humic acid as foliar spray applications); Kh (applying potassium humate as foliar spray applications); K_2_SO_4_ (applying potassium sulfate as foliar spray applications); 100 (applying 100% of gross irrigation water requirements); 80 (applying 80% of gross irrigation water requirements).

Table 5 showed the individual and interaction impacts of the examined irrigation levels, K, and H on the investigated parameters. The impacts of irrigation levels (100 and 80% GWR), H applications as (Hgd and Hsp), and K as (Kh and K_2_SO_4_) on carrots N are presented in (Fig. 1A). According to the obtained results of variance analysis, the effects of various individual and combination treatments on N contents were obvious. In general, the obtained results showed that by comparing the sole applications of K_2_SO_4_ and control (Hsp), The adoption of GWR80% led to a decrease in N contents of 31.9 and 22.9%, respectively, compared to GWR100%. However, when adopting GWR80%, N content was enhanced than GWR100% by applying sole soil applications of Hgd or a combined application of Hgd + Kh by 17.5 and 11.0%, respectively. While there was a non-significant effect by applying the combined applications of Hgd + K_2_SO_4_ and adopting GWR80% than GWR100%. On the other side, adopting GWR100% and applying the combined foliar applications of Hsp + Kh attained the maximum increase of N content (668 mg kg^−1^), although that significantly equaled the adoption of GWR100% x combined application of Hsp + K_2_SO_4_ (645 mg kg^−1^).

By comparing the examined applications under GWR100%, P content increased when applying combined applications of Hgd x foliar applications of K as (Kh or K_2_SO_4_), as can be seen in (Fig. 1B). Relative to control full irrigation treatment (GWR100%), P content increased by 23.0, 28.2,15.4, 63.6, 70.2, 28.5, and 45.4% for Kh, K_2_SO_4_, Hgd, Hgd + Kh, Hgd + K_2_SO_4_, Hsp + Kh, Hsp + K_2_SO_4_, respectively, while it was decreased by 4.5% under Hsp. Likewise, relative to control limited irrigation treatment (GWR80%), P contents increased by 48.9, 29.8, 36.2, 85.9, 93.5, 14.8, 57.9, and 80.2% for Kh, K_2_SO_4_, Hgd, Hgd + Kh, Hgd + K_2_SO_4_, Hsp, Hsp + Kh, Hsp + K_2_SO_4_, respectively. The results demonstrated that the highest P contents (53.4 mg kg^−1^), were obtained with GWR100% and applying combined foliar applications of Hgd + Kh, however, that significantly equaled the adoption of the combined application of Hgd + K_2_SO_4_ under the same irrigation level.

The results demonstrated that K has a unique role that can prevent serious injury to carrot plants under stressful conditions by increasing plant tolerance. Where, by comparing the control in (Fig. 1C), it was found that the adoption of GWR 80% had a significant difference in K contents compared to GWR100%. Therefore, when the limited irrigation was adopted, K contents increased by 27.7, 25.0, 18.2, 47.7, 37.5, 27.1, 43.0, and 45.0% for Kh, K_2_SO_4_, Hgd, Hgd + Kh, Hgd + K_2_SO_4_, Hsp, Hsp + Kh, Hsp + K_2_SO_4_, respectively, indicating that a limited irrigation water in these conditions increased K uptake. Additionally, it was found that the combined foliar applications of Hsp x foliar applications of K as (Kh or K_2_SO_4_) significantly increased K contents when adopting GWR100. Furthermore, GWR100% was pronounced with combined applications of Hsp + K_2_SO_4_ or GWR80% with combined applications of Hgd + Kh, for attaining the highest K content in the carrot root. However, applied combined applications of Hgd + Kh under water-limited treatment caused the greatest increase in K content to 735,4 (mg kg^−1^), an increase of 10.70% compared with that in Hgd + Kh under GWR100%.

**Figure 2:**
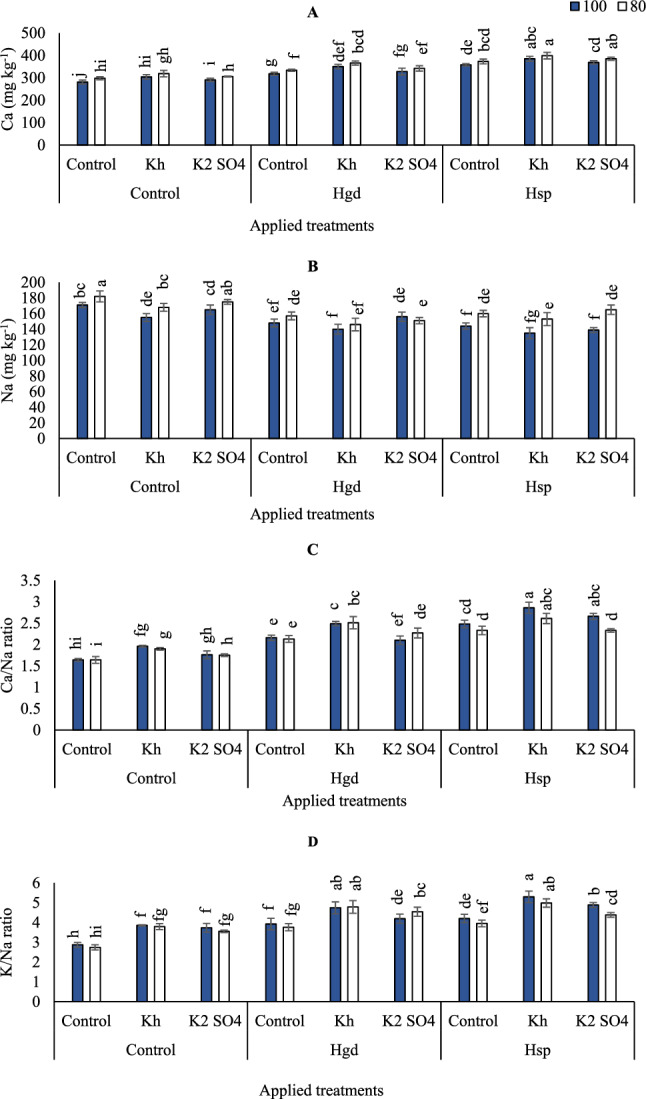
Influence of the sole or combined application of humic acid & potassium sources in the carrot plants under full and limited irrigation levels on Ca- calcium (**A**), Na- sodium (**B**), Ca/ Na ratio (**C**), and K/ Na ratio (**D**). The illustrated values in the figures are the average of the two growing seasons of 2019/2020 and 2020/2021. Vertical bars represent ± standard error (SE) of the means. Bars with different letters are statistically significant at *p* ≤ 0.05. Abbreviations: Control (spray with pure water); Hgd (applying humic acid as soil application); Hsp (applying humic acid as foliar spray applications); Kh (applying potassium humate as foliar spray applications); K_2_SO_4_ (applying potassium sulfate as foliar spray applications); 100 (applying 100% of gross irrigation water requirements); 80 (applying 80% of gross irrigation water requirements).

The Ca content in carrot root increased from 281 (mg kg^−1^) for the control to 385 (mg kg^−1^) in GWR100% treatment and applying Hsp + Kh (Fig. 2A). Likewise, adopting GWR80% of irrigation level and applying combined applications of Hsp + Kh or Hsp + K_2_SO_4_ were significantly equaled in attaining the greatest increase in Ca content under these stressful conditions, reaching about 399 and 385 (mg kg^−1^), respectively.

Based on the results of variance analysis, the individual and interaction impacts of examined irrigation levels, K, and H obviously affected Na content mainly by regulating water status. However, the dual interaction effects of K applications and irrigation were insignificant. The content of Na in carrot root was increased by adopting GWR80% compared to GWR100%, (Fig. 2B). Although foliar applications of K_2_SO_4_ in combination with Hgd increased Na content when adopting GWR100%. However, when adopting GWR80%, Na content attained the greatest increase by applying the sole foliar applications of K_2_SO_4_ (175 mg kg^−1^), which significantly equaled applying tap water (control). Compared irrigation levels under different examined applications, Na content was increased under GWR80% by 4.0, 8.4, 6.1, 6.1, 3.6, 11.1, 13.3, and 18.7% for control, Kh, K_2_SO_4_, Hgd, Hgd + Kh, Hsp, Hsp + Kh, Hsp + K_2_SO_4_, respectively.

The impacts of irrigation levels (100 and 80% GWR), H applications as (Hgd and Hsp), and K as (Kh and K_2_SO_4_) on the Ca /Na ratio in carrots are presented in (Fig. 2C). Depending on the results of variance analysis, the individual and interaction impacts of examined irrigation levels, K, and H obviously affected Ca /Na ratio. However, the individual impact of irrigation or the dual interaction effects of K applications and irrigation were insignificant. By comparing the examined treatments, occurred that Ca /Na ratio reached the peak by applying the combined foliar applications of Hsp + Kh by 2.86 and 2.61 under GWR100&80%, respectively. While those in the Hsp + K_2_SO_4_ treatment mostly reached a peak either (2.66), under GWR100%.

To maximize K /Na ratio in the carrot root, either Hgd combined with Kh or Hsp + Kh was applied to provide a protracted, under both irrigation levels (Fig. 2D). According to the results of variance analysis, the individual and interaction impacts of examined irrigation levels, K, and H obviously affected K /Na ratio. However, the individual impact of irrigation levels or the dual interaction effects of K applications and irrigation were insignificant. The results indicated that applied tap water under GWR100&80% led to a lower K /Na ratio (2.87 and 2.74, respectively). The highest K /Na ratio was obtained with GWR100&80% irrigation levels and applying foliar applications of Hsp + Kh. The following highest K /Na ratio was seen with the same irrigation level by using a combined application of Hgd + Kh.

**Figure 3:**
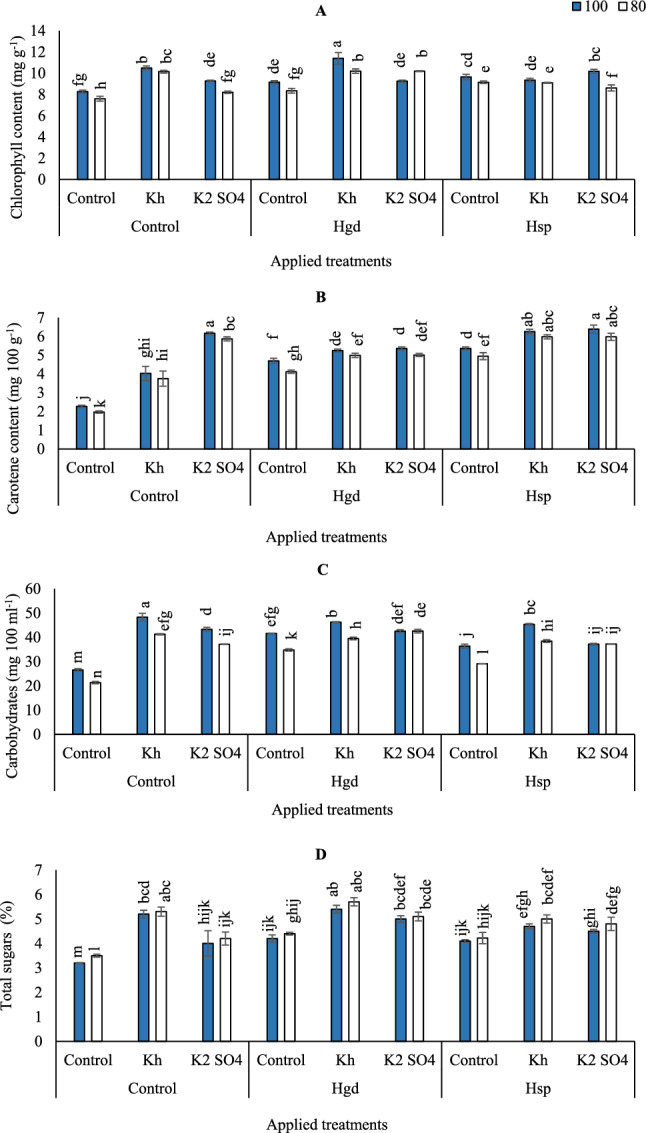
Influence of the sole or combined application of humic acid & potassium sources in the carrot plants under full and limited irrigation levels on chlorophyll (**A**), carotene (**B**), carbohydrates (**C**), and total sugar (**D**). The illustrated values in the figures are the average of the two growing seasons of 2019/2020 and 2020/2021. Vertical bars represent ± standard error (SE) of the means. Bars with different letters are statistically significant at *p* ≤ 0.05. Abbreviations: Control (spray with pure water); Hgd (applying humic acid as soil application); Hsp (applying humic acid as foliar spray applications); Kh (applying potassium humate as foliar spray applications); K_2_SO_4_ (applying potassium sulfate as foliar spray applications); 100 (applying 100% of gross irrigation water requirements); 80 (applying 80% of gross irrigation water requirements).

To increase the content of chlorophyll during the whole growth period, the carrot plant significantly influenced its chlorophyll formation and metabolic functions depending on irrigation quantities and K nutrition source. Therefore, chlorophyll content in the carrot leaves was enhanced by adopting GWR 100% compared to GWR 80%, (Fig. 3A). Although the sole applications of Hgd + Kh significantly improved chlorophyll content when adopting GWR80%, indicating the crucial role that K combined with humic play in plant-water relations under limited irrigation conditions. In the full irrigation (GWR100%) under different examined applications treatments, chlorophyll content increased compared to GWR 80% by 8.8, 3.5, 13.1, 9.7, 11.8, 5.5, 2.8, and 18.1% for control, Kh, K_2_SO_4_, Hgd, Hgd + Kh, Hsp, Hsp + Kh, Hsp + K_2_SO_4_, respectively. While it was increased under GWR80% compared to GWR100% by 10.3% for Hgd + Kh treatment. Generally, full irrigation level (GWR100%) was pronounced with the combined soil Hgd x foliar applications of Kh for attaining the highest chlorophyll content (11.4 mg g^−1^).

On the other hand, compared to the control treatments in (Fig. 3B), the contents of the carotene were significantly increased by adopting GWR100% compared to GWR80%, except for the applications of Kh, Hgd + Kh, Hgd + K_2_SO_4_, Hsp + Kh, and Hsp + K_2_SO_4_. The highest carotene contents were recorded by applying combined foliar applications of Hsp + (Kh or K_2_SO_4_) and adopting GWR100&80%, although that significantly equaled GWR100% x sole application of K_2_SO_4_. While the lowest carotene content was obtained under control GWR80% irrigation level (1.97 mg 100 g^−1^).

According to the results of variance analysis (Table 5), the individual and interaction impacts of examined irrigation levels, K, and H obviously affected carbohydrate content. However, the dual interaction effects of H applications and irrigation were insignificant. As can be seen in **(**Fig. 3C), adopting GWR80% of irrigation level decreased the carbohydrate content in the carrot root compared to GWR100%, except for the combined applications of Hgd + K_2_SO_4_ or Hsp + K_2_SO_4_. Moreover, the maximum increase of carbohydrates was obtained for the sole foliar applications of Kh when adopting GWR100%. The highest carbohydrate contents (48.3 mg 100 mL^−1^) were obtained by applying sole foliar applications of Kh and adopting GWR100. Likewise, adopting GWR80% without any auxiliary applications attains the lowest carbohydrate content (21.3 mg 100 mL^−1^).

Relative to control full irrigation treatment (GWR100%), total sugars in the carrot root in (Fig. 3D) increased by 62.5, 25.0, 31.3, 68.8, 56.3, 28.1,46.9 and 40.6% for Kh, K_2_SO_4_, Hgd, Hgd + Kh, Hgd + K_2_SO_4_, Hsp, Hsp + Kh, Hsp + K_2_SO_4_, respectively. Likewise, relative to control GWR80%, total sugars increased by 51.4, 20.0, 25.7, 62.9, 45.7, 20.6, 42.9, and 37.1% for Kh, K_2_SO_4_, Hgd, Hgd + Kh, Hgd + K_2_SO_4_, Hsp, Hsp + Kh, Hsp + K_2_SO_4_, respectively. Collectively, spraying Kh applied as combined with soil application of Hgd under GWR100& 80%, resulted in significantly higher values than the other treatments (5.4 and 5.7%, respectively), although that significantly equaled GWR80% x sole application of Kh (5.3%).

### Impacts of the H and K applications on carrots agronomic traits under full and limited irrigation levels.

**Figure 4:**
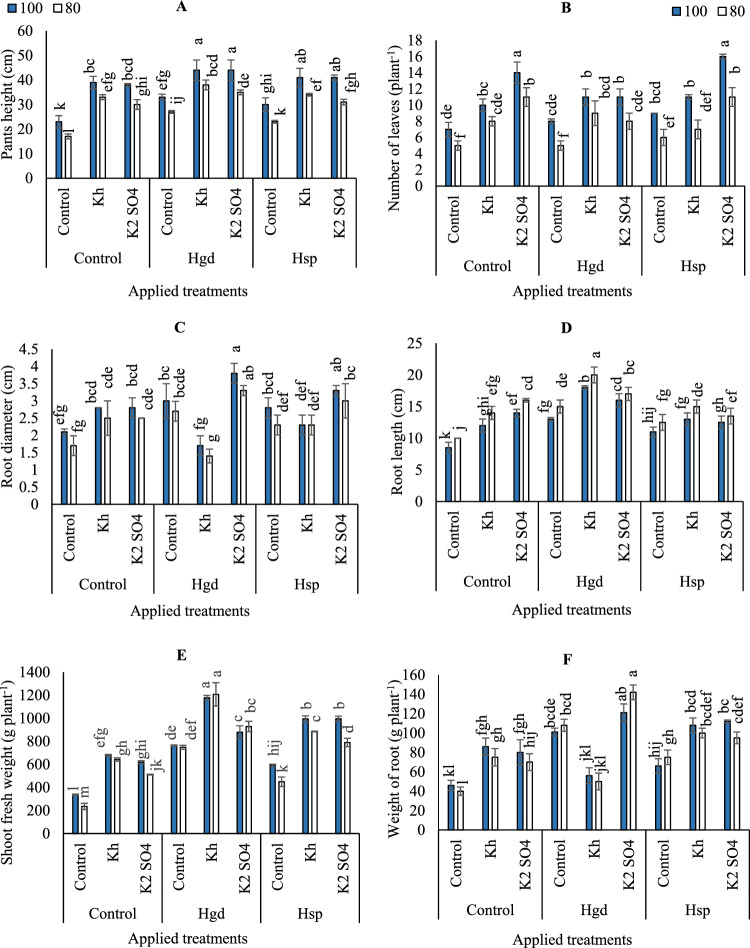
Influence of the sole or combined application of humic acid & potassium sources in the carrot plants under full and limited irrigation levels on carrot pants height (**A**), number of leaves per plant (**B**), root diameter (**C**), root length (**D**), shoot fresh weight (**E**), and weight of root (**F**). The illustrated values in the figures are the average of the two growing seasons of 2019/2020 and 2020/2021. Vertical bars represent ± standard error (SE) of the means. Bars with different letters are statistically significant at *p* ≤ 0.05. Abbreviations: Control (spray with pure water); Hgd (applying humic acid as soil application); Hsp (applying humic acid as foliar spray applications); Kh (applying potassium humate as foliar spray applications); K_2_SO_4_ (applying potassium sulfate as foliar spray applications); 100 (applying 100% of gross irrigation water requirements); 80 (applying 80% of gross irrigation water requirements).

Based on the results of variance analysis, the individual and interaction impacts of examined irrigation levels, K, and H obviously affected plant height. However, the dual interaction effects of H applications and irrigation or K applications and irrigation were insignificant. The results in (Fig. 4A) indicated that the adoption of GWR80% led to a decrease in carrot plant height. Therefore, when the limited irrigation was adopted, plant height values decreased by 35.3, 18.2, 26.7, 22.2, 15.8, 25.7, 30.4,20.6 and 32.3% for control, Kh, K_2_SO_4_, Hgd, Hgd + Kh, Hgd + K_2_SO_4_, Hsp, Hsp + Kh, Hsp + K_2_SO_4_, respectively, indicating that apply full irrigation water under these conditions increased plant height. The highest plant height was obtained with GWR100% irrigation level and applying the applications of Hgd or Hsp applied as combined with foliar applications of (Kh or K_2_SO_4_). The lowest plant height was seen under control at GWR80%.

By comparing the control in (Fig. 4B), it was found that the adoption of GWR80% irrigation level had a significant difference in the number of carrot leaves compared to GWR100%. Additionally, this impact has remained unchanged under the various examined treatments, except for the sole foliar applications of Kh or under Hgd + Kh treatment. In addition, the maximum increase in the number of carrot leaves was obtained for the sole foliar applications of K_2_SO_4_ when adopting GWR100% (14), that were significantly equaled GWR100% x the combined applications of Hsp + K_2_SO_4_ (11).

As can be seen in **(**Fig. 4C), it was found that the combined foliar applications of Hgd x foliar applications of Kh significantly decreased the diameter of carrot root when adopting GWR100& 80%, although that significantly equaled the control GWR100& 80% without any auxiliary applications. On the other side, both combined applications of Hsp + K_2_SO_4_ or Hgd + K_2_SO_4_ were pronounced for attaining the highest root diameter values under GWR100&80%.

On the other hand, the superiority of Hgd applications with limited irrigation (GWR80%) is still pronounced compared to Hsp causing increases in carrot root length, shoot fresh weight and the weight of carrot roots as can be seen in (Fig. 4D,E,F). According to the results of variance analysis, the individual and interaction impacts of the examined irrigation levels, K, and H on root length were significant; however, the interaction impacts of irrigation + K or irrigation + H were not significant. Similarly, the individual and interaction impacts of the examined irrigation levels, K, and H on shoot fresh weight were significant, however, the individual impacts of irrigation or the interaction impacts of irrigation + K on the weight of carrot roots were not significant. The results indicated that the highest increase in the carrot root length (20 cm), was obtained for the combined application of Hgd + Kh when adopting GWR80%. Likewise, the adoption of combined application of Hgd + Kh applications under GWR80% attained the highest shoot fresh weight (1207 g plant^−1^), although that significantly equaled GWR100% x combined application of Hgd + Kh (1177 g plant^−1^). Moreover, the adoption of combined application of Hgd + K_2_SO_4_ applications under GWR80% attaining the highest weight of carrot roots (142 g plant^−1^), although that significantly equaled GWR100% x combined application of Hgd + K_2_SO_4_ (121 g plant^−1^).

### Impacts of the H and K applications on carrots yield and Iwue under full and limited irrigation levels

**Figure 5:**
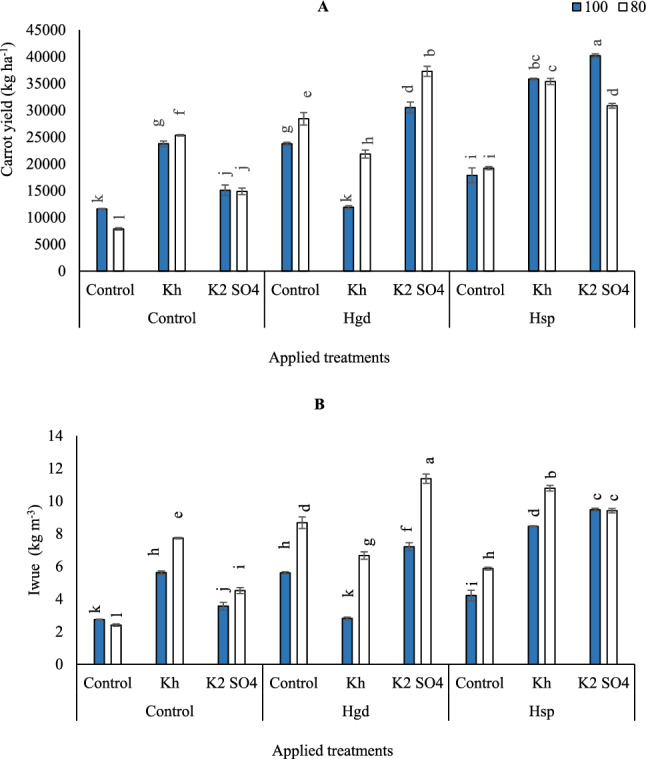
Influence of the sole or combined application of humic acid & potassium sources in the carrot plants under full and limited irrigation levels on carrot yield (**A**) and Iwue- irrigation water use efficiency (**B**). The illustrated values in the figures are the average of the two growing seasons of 2019/2020 and 2020/2021. Vertical bars represent ± standard error (SE) of the means. Bars with different letters are statistically significant at *p* ≤ 0.05. Abbreviations: Control (spray with pure water); Hgd (applying humic acid as soil application); Hsp (applying humic acid as foliar spray applications); Kh (applying potassium humate as foliar spray applications); K_2_SO_4_ (applying potassium sulfate as foliar spray applications); 100 (applying 100% of gross irrigation water requirements); 80 (applying 80% of gross irrigation water requirements).

According to the results of variance analysis, the individual and interaction impacts of examined irrigation levels, K, and H obviously affected carrot yield. Compared to the examined irrigation level in control treatment without any auxiliary applications, carrot yield was increased by adopting GWR100% of irrigation level compared to GWR80%, (Fig. 5A). Although there was an improvement by applying auxiliary applications as sole or combined forms when adopting GWR80%. Relative to control full irrigation treatment (GWR100%), carrot yield increased by 105.0, 30.0, 104.8, 2.7, 163.1, 54.1, 208.9, and 246.4% for Kh, K_2_SO_4_, Hgd, Hgd + Kh, Hgd + K_2_SO_4_, Hsp, Hsp + Kh, Hsp + K_2_SO_4_, respectively. Likewise, relative to control limited irrigation treatment (GWR80%), carrot yield increased by 222.2, 89.2, 261.4, 178.0, 374.0, 144.3, 349.9, and 292.3% for Kh, K_2_SO_4_, Hgd, Hgd + Kh, Hgd + K_2_SO_4_, Hsp, Hsp + Kh, Hsp + K_2_SO_4_, respectively. The highest carrot yield was observed by applying the foliar applications of Hsp + K_2_SO_4_ under GWR100% (40,183 kg ha^−1^). Likewise, when adopting the GWR80% irrigation level without any auxiliary applications, the carrot yield attained the lowest recorded value (7865 kg ha^−1^). Furthermore, the combined applications of Hgd + Kh were found to have decreased carrot yield, particularly under GWR 100%.

As can be seen in (Fig. 5B), under limited irrigation water, Iwue slightly decreased significantly in control treatment by 2.4 kg m^−3^, a decrease of 14.2% compared to that in GWR100%. By adopting the GWR80% irrigation level, Iwue increased from 2.4 (kg m^−3^) for the control GWR80% without any auxiliary applications to 11.4 (kg m^−3^) for Hgd + K_2_SO_4_. Additionally, by adopting GWR80% Iwue increased by 137.0% in Hgd + Kh and by 57.9% in Hgd + K_2_SO_4_ compared with that in GWR100%. The lowest Iwue values 2.81 (kg m^−3^) were recorded by applying combined foliar applications of Hgd + Kh and adopting GWR100%, although that significantly equaled the control of GWR100& 80% without any auxiliary applications. The lowest Iwue values were in control GWR80%, followed by control GWR100% (2.81 kg m^−3^) or under the same irrigation level (GWR100%) × (Hgd + Kh) at 2.81 kg m^−3^.

## Discussion

Efficient water management has become imperative for water conservation in many regions worldwide. Implementing appropriate irrigation scheduling and fertigation techniques can help reduce water loss and increase Iwue.

In our study, we observed significant yield reductions in carrot crops subjected to limited irrigation at GWR80%. This indicates that under GWR80%, the available water for carrot plants is critically reduced, resulting in decreased solubility and uptake of essential macronutrients such as N and P (Fig. 1A,B), and an increase in Na uptake (Fig. 2B). Notably, P plays a crucial role in root growth and architecture^49–51^. While N and P are essential for chlorophyll and carbohydrate synthesis, as depicted in (Fig. 3A,C). The limited availability of these nutrients leads to root elongation in search of water and nutrients, which subsequently results in reduced plant height, root diameter, root length, shoot fresh weight, and weight of carrot roots. Ultimately, this decreases the efficiency of the carrot plant and reflects in reduced yield and Iwue under these conditions. These findings align with previous studies, such as that of Ali^52^, which identified the sensitivity of carrot plants to water stress, especially during the initial and developmental stages. Additionally, Abdel-manly^53^ suggested avoiding water stress during the carrot growing season and, at worst, setting an irrigation threshold of 40% soil water deficiency to mitigate the severe impacts of water stress on carrot yield.

Based on this premise, our study hypothesized that providing plants with supplemental applications would enable them to overcome and improve their defense mechanisms against the adverse effects of water stress.

Regarding these supplemental applications, our results indicate that under full irrigation, adopting combined applications of humic acid through foliar applications (Hsp) along with either Kh or K_2_SO_4_ was beneficial. Conversely, under limited irrigation at GWR80%, sole and combined applications of humic acid through soil drenching (Hgd) with K_2_SO_4_ showed better results in increasing carrot yield. We postulated that under GWR100% irrigation, there is a higher stomatal opening in plant leaves^54,55^. Therefore, applying foliar applications of Hsp in conjunction with potassium applications, either as Kh or K_2_SO_4_, enhances nutrient absorption due to the synergistic benefits of potassium and humic acid applications. Particularly, Hsp facilitates the direct delivery of sufficient nutrients to the production sites (plant leaves), compensating for the reduced efficiency of the carrot roots under these conditions^47^, which subsequently leads to increased nutrient absorption as shown in (Figs. 1A,C and 2A,C,D). Furthermore, this results in improvements in physiological processes such as transpiration and water storage in carrot leaves, ultimately leading to improved carrot yield (Fig. 4A) under these conditions. On the other hand, under GWR80%, the sole application of Hgd was more effective in increasing carrot yield compared to Hsp. In this scenario, we hypothesized that plants tend to reduce their activities, including decreased transpiration rates and increased stomatal closure, as a protective mechanism to cope with water stress and maintain their water status. Under these conditions, the root system becomes the controller of plant activities^47^. Hence, the application of Hgd offers several benefits, including improved soil water retention capacity, enhanced nutrient uptake in both shoots and roots, increased vegetative growth, and yield, as well as carbohydrate and carotenoid content, which is consistent with the findings of previous studies^14,56^.

In contrast, we observed negative effects on carrot yield when adopting the combined applications of Hgd + Kh, especially under full irrigation conditions. We attribute this to the high doses of humic substances resulting from the simultaneous application of Hgd + Kh under favorable conditions. To clarify, the foliar application of Kh appeared to provide significant amounts of plant nutrient requirements, compensating for the reduced efficiency of the carrot roots, particularly under GWR100%. On the other hand, while the application of Hgd improved soil organic matter contents (Table 4) and soil physical properties^10,57^, formed chelate components with cationic metals^58,59^, and enhanced nutrient metabolism and photosynthesis^8,60^, it seemed to primarily promote rapid growth of vegetative plant organs rather than reproductive plant organs. This led to a shortened storage period compared to the vegetative period, resulting in reduced root diameter and weight (Fig. 4C,F), but increased plant height, number of leaves, and shoot fresh weight (Fig. 4A,B,E). These outcomes align with the findings of previous studies^61^ indicating that high doses of humic substances can improve soil physical characteristics but have uncertain effects on chemical soil characteristics and crops^28^. Furthermore, Raheem et al.^62^ demonstrated that increasing the application rate of humic acid resulted in the lowest total yield of lettuce, and Maibodi et al.^63^ found that only 100 mg l^−1^ of humic acid improved various characteristics of *perennial ryegrass*. Asri et al.^64^ showed that tomato fruit weight, diameter, and yield increased with increasing rates of humic acid up to a certain threshold, beyond which negative effects emerged. Similar findings have been reported by^10,65^ highlighting the variability of organic amendments' impact on crop yield, which is influenced by environmental factors, soil conditions, the composition of the amendment, and crop type. Therefore, our results emphasize the importance of careful fertigation management, particularly when applying organic amendments under full irrigation conditions. This approach promotes accelerated production and storage processes over vegetative processes, resulting in increased carrot yield.

Based on our findings, the adoption of combined applications of Hgd + K_2_SO_4_ under GWR80% improved carrot yield and achieved the highest Iwue values. We hypothesize that when implementing limited irrigation, plants experience some degree of water stress, which triggers a series of physiological responses. These reductions in water availability seem to enhance root efficiency^47^. While negatively impacting yield under control treatments. However, applying Hgd improves soil water and nutrient status by increasing nutrient availability, leading to improved water and nutrient absorption from the rhizosphere, as observed in available K values (Table 4). This, in turn, increases agronomic traits (Fig. 4C,F), carrot yield (Fig. 5A), and enhances plant tolerance to water stress. These findings align with previous studies^14,66^, which have shown the indirect effects of humic acids on soil properties such as aggregation, aeration, permeability, water-holding capacity, micronutrient transport, and availability^67–69^ , as well as the direct effects on photosynthesis, plant growth, crop performance, and nutrient uptake rate^10,70^. Furthermore, the foliar application of K_2_SO_4_ provides significant amounts of potassium, which contributes to cell membrane integrity through osmotic adjustment, regulates stomatal movement, catalyzes cell division, enhances yield and quality, facilitates carbohydrate transformation, and promotes sugar synthesis^71–74^. Additionally, humic substances and K_2_SO_4_ enhance the exudation of organic acids by plant roots (Table 4), influencing root length, carrot size, and membrane integrity through osmotic adjustment, thus affecting root fresh weight and yield^14,75^. These findings are consistent with Rose et al.^28^, who demonstrated that the combined application of humic and mineral fertilizers forms complexes that release nutrients slowly, leading to increased yield. Consequently, the combined applications result in higher yields with reduced irrigation quantities, leading to the highest Iwue for carrots under these conditions.

## Conclusion

This research highlights the positive effects of supplying stressed carrot plants with auxiliary foliar applications of potassium and humic amendments, leading to improved nutrient uptake and carrot yield. However, the combined application of foliar potassium humate and soil amendments of humic resulted in a decrease in carrot yield, regardless of whether full or limited irrigation levels were applied. These findings challenge the notion that organic applications always enhance yield and suggest that the impact is influenced by factors such as the specific fertigation technique and the quantities of humic substances applied. Further studies are needed to explore this impact on other crops.

For regions facing water scarcity, it is recommended to adopt combined foliar applications of potassium sulfate and soil amendments of humic at 80% of the gross irrigation water requirements as auxiliary treatments for water-stressed carrot plants. These combined applications have shown the potential to improve nutrient uptake, carrot yield, and mitigate the effects of water stress. Furthermore, they enable a higher increase in water use efficiency for carrot crops.

## Data Availability

The presented datasets during the current study available from the corresponding author on reasonable request.
